# Transactions of the Medical Society of the State of Missouri, at Its Second Annual Session, April, 1852

**Published:** 1852-11

**Authors:** 


### EDITORIAL.

*Transactions of the Medical Society of the State of Missouri, at its second Annual Session, April,* 1852.

THE annual address of the President, Wm. M. McPheaters, M. D., is devoted to the subject of medical reform. While he admits that the standard of professional education is much lower here, than in Europe, where the strong arm of law is exerted to protect the man of science, and crush the pretender; he shows conclusively, that legal enactments for the protection of the medical profession, equally with those for the establishment of religious systems, are inconsistent with the genius and spirit of our institutions; that the only line of distinction that can be drawn, between the faithful, educated physician, and the arrogant, illiterate quack, must be the plain distinction between ignorance and learning; between empiricism and science. Medical schools are shown to be dependent entirely upon, and consequently the standard of requirements for graduation capable of being regulated by, the profession. The responsibility is, therefore, not with the schools, but with the mass of physicians in whose offices students receive, or should receive, a large part of their medical education: but, in order to accomplish any thing valuable, there must be concert of action.—The machinery of reform is already at work in our National, State, County, and local societies. Let the recommendations of our National Congress be regarded as laws, for the governance of State and subordinate associations, and the work will soon be accomplished.

The report of the Committee on Surgery, from the pen of Prof. Charles C. Pope, is well written, and contains a digest of the recent improvements in that branch, both in this country and Europe. After noticing Dr Sims' method of treating vesico-vaginal fistula, he says:

A most ingenious, and, it seems to me, valuable improvement on Dr. Sims' suture apparatus, has been mdae by our townsman, Mr. J. Thompson. Wishing to obviate the tediousness and difficulty of application of Dr. S.'s contrivance, he has imagined a mechanism by which the wires are secured in the form of small teeth, to the posterior clamp, which is likewise furnished with two small ratchets, the whole intended to be received in corresponding apertures in the anterior clamp; when, by simple and gentle pressure, the two are approximated and retained. The edges can be freshened either before or after the application of the suture.—While possessing the same retentive power as the suture of Dr. Sims, it would seem to have the advantage in being more easily applied and removed. By the aid of certain appliances, it is easy to effect both movements. A slight modification adapts the contrivance to longitudinal, as well as the transverse fistulæ. Mr. Thompson has extended his idea to sutures adapted to hare lip, staphyloraphy, hypospadias, ruptured perineum, and other cases, whose beautiful application must be seen to be properly appreciated.

We sincerely hope that the new impetus and improved methods given to these cases of vesico-vaginal fistula, will be attended by the best results in extending the domain of surgery, and in affording comfort and happiness to many hitherto incurable and deplorable patients.

Within the last fortnight, we have operated on a case of vesico-vaginal fistula, besides the one already referred to. We made trial of the method of Dr. Sims. The position which he recommends is certainly most advantageous for both sight and manipulation, and possesses all the benefit claimed for it by its author. Through the large opening, one inch and a half in length, by three quarters of an inch in width, and situated transversely at the neck of the bladder, we could easily look into that empty viscus, and distinctly trace the rugæ of its sides and fundus. The catheter also fully answered our expectations. A difficulty of no inconsiderable magnitude, with which we had to contend in the management of this case, was an almost complete co-arctation of the vagina, immediately posterior to the fistulous orifice. We felt inclined to attempt first, a closure of the abnormal opening of the bladder, with the intention, if successful, of subsequently endeavoring to overcome the vaginal stricture. After having thoroughly freshened the borders of the fistula, we succeeded in applying the suture apparatus of Dr. Sims, most satisfactorily; but whether from the proximity of the stricture, or from the inodular tissue easily taking on ulceration; whether a too close proximity of the clamps had been effected, or from other and all these causes combined, we failed in obtaining more than a very partial success, the opening having been closed in about a fourth of its extent.

The report on obstetrics contains the opinions of the Committee on questions now exciting an interest among obstetricians. On the subject of anæsthesia in midwifery, after meeting objections urged against it, and enumerating some of its positive advantages; they hold the following language:

Although we believe that chloroform is a harmless agent, when properly administered, we do not advocate, nor do we practice, its indiscriminate use in midwifery. We think that there are contra-indications to its use. In labors altogether favorable, there can be no urgent reason to use it. But when the labor is unusually severe and protracted, the physical suffering demands alleviation on account of itself, and because it is apt to produce pathological conditions. In such cases, it is not necessary to push chloroform so far as to produce unconsciousness.

In instrumental labors, it is not necessary to carry the inhalation any further; nor is it in cases of ordinary turning. It is only in cases of difficult turning, that it must be used in a bolder manner, to overcome the contractions of the womb, which would otherwise render the operation almost impracticable.

On the nature of puerperal fever, after an interesting examination of the causes producing it, and the manner in which the circulating fluids may become vitiated, they draw the following inferences.

From the evidence before us, derived from a careful study of the recorded experience of others, and our own observations, we hold these propositions to be true:

I. Puerperal fever is an essential fever, the source of which is to be found in the vitiated state of the blood.

II. Such vitiation may exist in a mild form, or a more deadly form, without any inflammation.

III. Most frequently, however, the condition of the blood produces inflammation in some of the organs.

IV. Such inflammations may be in the peritoneum, the mucous membrane of the bowels, in the veins of the uterus, in the proper tissue of the uterus, or its appendages, or in any of these combined.

V. As in other cases of puerperal affection, secondary inflammation, followed by puerperal deposits, may arise, in the thorax, in the joints, in the eye, or in the muscles.

The practical propositions are these:

I. That as puerperal fever does exist without inflammatory complications, such cases do not require the use of the lancet or other direct depletives.

II. That owing to the quantity of the poison received into the circulation, or to the feeble habit of the body, which prevents a due resistance to it, or to both of these combined, inflammation sometimes arises, which is of so low a type that depletives are to be used with much caution.

III. That when open inflammation arises, it then becomes the predominant element of the disease, and the loss of blood is borne with more toleration in inflammation, than even in a state of health. The lancet is then to be used boldly and decisively, as also its adjuvants, calomel, opium, &c.

The report on the domestic adulteration of drugs, shows that mercurial ointment and blue mass has been so largely adulterated as in many instances to be entirely inert. The pulverized vegetable medicines are also very liable to adulteration, and that too, by domestic dealers. The report concludes by recommending the adoption of measures to secure legal interference.

The transactions contain also an essay on medical education, by A. Hammer, M. D.; an essay on erysipelas, by J. P. Vaughan, M. D.; a report on the topography of the county of Boone, by J. Wilcox, M. D., and a communication on bilious fever, by H. Schoeneick, M. D.

The volume does honor to the society. J.

